# Genomic Sequencing and Analysis of Enzootic Nasal Tumor Virus Type 2 Provides Evidence for Recombination within the Prevalent Chinese Strains

**DOI:** 10.3390/vetsci11060248

**Published:** 2024-06-02

**Authors:** Yixuan Li, Jingyi Niu, Yiyu Liu, Yu Dai, Hongbo Ni, Jinliang Wang, Rendong Fang, Chao Ye

**Affiliations:** 1 Joint International Research Laboratory of Animal Health and Animal Food Safety, College of Veterinary Medicine, Southwest University, Chongqing 400715, China; lyx03014525@163.com (Y.L.); 13220247782@163.com (J.N.); liuyiyu0321@163.com (Y.L.); dy010126@163.com (Y.D.); rdfang@swu.edu.cn (R.F.); 2 College of Veterinary Medicine, Qingdao Agricultural University, Qingdao 266109, China; 3 Shandong Binzhou Animal Science & Veterinary Medicine Academy, Binzhou 256600, China

**Keywords:** enzootic nasal tumor virus type 2, phylogenetic analysis, recombination, genomic analysis

## Abstract

**Simple Summary:**

Enzootic nasal tumor virus type 2 (ENTV-2), the causative agent of enzootic nasal adenocarcinoma (ENA), is a highly lethal retrovirus of goats. To investigate the genetic variation of ENTV-2 in China, we conducted genomic sequencing and a comprehensive analysis of an emerging ENTV-2 strain CQ2 from goats in Chongqing, China. Our phylogenetic analysis revealed that CQ2 belonged to a distinct sublineage that was different from the previously reported ENTV-2 strain CQ1 in Chongqing. Furthermore, genomics analysis indicated that CQ2 was a previously unreported recombinant strain. These findings contribute to our understanding of the evolution of ENTV-2 in China and provide novel insights into its variation.

**Abstract:**

In recent years, the clinical cases of ENTV-2 infection have increased and become prevalent in several provinces of China. In this study, we reported the occurrence of ENTV-2 in one goat farm in Chongqing, southwest China. The complete genome of an emerged ENTV-2 isolate (designated as CQ2) was sequenced with 7468 bp in length. Phylogenetic analysis revealed that ENTV-2 consisted of two main lineages. Lineage 1 was composed of Chinese strains and could be subdivided into five sublineages. CQ2 and the other six recent isolates from China were clustered in sublineage 1.5; however, CQ2 was significantly different from the other six isolates. Furthermore, recombination analysis suggested that CQ2 might be a recombinant variant derived from sublineage 1.5 and sublineage 1.2 strains, with the recombination region in areas of *pro* and *pol* genes. In conclusion, we sequenced and analyzed the complete genome of a potential ENTV-2 recombinant, which may contribute to our understanding of the genetic variation and evolution of ENTV-2 in China.

## 1. Introduction

Enzootic nasal adenocarcinoma (ENA) is a contagious disease characterized by neoplastic transformation of secretory epithelial cells in the respiratory tract of sheep and goats, resulting in substantial economic losses to the goat and sheep-breeding industry [1]. It is currently known that ENA is associated with enzootic nasal tumor virus (ENTV), although the role of ENTV in the pathogenesis of ENA has yet to be resolved due to the inability to culture the virus [2].

ENTV is composed of two distinct viruses, i.e., the enzootic nasal tumor virus of sheep (ENTV-1) and the enzootic nasal tumor virus of goats (ENTV-2) [3]. In terms of taxonomy, ENTV belongs to the genus *Betaretrovirus* under the *Retroviridae* family, which has the characteristics of B-type and D-type oncoviruses. The genome of ENTV is a single positive-stranded RNA of about 7.5 kb [4], which has the same basic canonical structure as other retroviral genomes, including four open reading frames (ORFs), 5′ and 3′ untranslated regions (UTRs), and long terminal repeats (LTRs) in the beginning and end of the sequence. The ORF region of ENTV encodes several functional proteins and consists of four overlapping genes, that is, *gag*, *pro*, *pol*, and *env* [5].

Since the first report of ENA in Germany in 1939 [1], except for Australia and New Zealand, the occurrence of this disease has been reported in the United Kingdom, France, Spain, Italy, Turkey, India, Brazil, and other countries worldwide [5,6,7,8]. In China, ENA was first reported in Inner Mongolia in 1975 [9]. In recent years, ENA has been reported in several provinces of China, including Guangdong, Guangxi, Chongqing, Sichuan, Yunnan, Fujian, Hunan, Shaanxi, and Anhui [3,10,11,12,13,14,15], indicating the widely spread of ENTV in China. Meanwhile, phylogenetic analysis of ENTV in goats (ENTV-2) in China indicated that novel ENTV-2 strains with distinct genome characteristics continuously appeared in recent years [10,16], suggesting that ENTV-2 has a relatively high variability and deserves continuous attention. In addition, the genetic variation in retroviruses is even more complex due to their ability to integrate the host genome [17]. The most notorious retrovirus, human immunodeficiency virus (HIV), has exhibited a high recombination rate in evolution. As a result, recombination is a major force in HIV evolution [18,19]. Although recombination is the main source of the retrovirus variation, the recombination of ENTV-2 has not been reported yet.

In this study, we detected the infection of ENTV-2 from clinical samples of goats with suspected ENA in Chongqing and obtained the complete sequence of one ENTV-2 sample (designated as CQ2) by RT-PCR and the subsequent Sanger sequencing. Phylogenetic analysis based on the complete genome of ENTV-2 showed that the virus was divided into two lineages: the Chinese strains belonged to lineage 1, while the strains from the UK belonged to lineage 2. In general, the genetic evolution of ENTV-2 displayed a geographical distribution pattern. Furthermore, among Chinese strains, ENTV-2 exhibited diversity and could be divided into five sublineages. In addition, comparative genomics analysis suggested that gene recombination may have occurred in the ENTV-2 genome. Noticeably, recombination analysis further indicated that an unreported recombination event could occur between different ENTV-2 strains. It was found that CQ2 might be a recombinant variant with the CHN4 isolate (Sichuan, China) as the minor parent and the AH2 isolate (Anhui, China) as the major parent, and with the recombination region in areas of pro and pol genes. In conclusion, like other retroviruses, ENTV-2 showed high variability and may generate a natural recombinant strain during clinical infection and evolution.

## 2. Materials and Methods

### 2.1. Clinical Samples

With the owners’ consent, a total of ten nasal swab samples were collected from goats with suspected ENA in Chongqing, China. The nasal fluids were preserved in a −80 °C freezer and prepared for subsequent RNA extraction. The sampling procedures were approved by the Institutional Animal Care and Use Committee (IACUC) of Southwest University, Chongqing, China (IACUC-20221031-08).

### 2.2. RNA Extraction

Nasal fluids were subjected to RNA extraction by using TRIzol Reagent (Life Technologies Carlsbad, CA, USA) as described previously [10]. Then, the RNA was digested with DNase I (TIANGEN, Beijing, China) and subsequently used as a template for one-step RT-PCR analysis. Concentrations of RNA ranged from 100 to 200 ng/μL.

### 2.3. The Complete Genome Amplification of ENTV-2 CQ2

A one-step RT-PCR Kit (Takara, Dalian, China) was used for one-step RT-PCR with a final reaction volume of 25 μL. The reaction mixture contained 12.5 μL of 2 × one-step buffer, 1 μL of PrimeScript 1-step enzyme mix, 1 μL of FQ-RT Primer Mix (TIANGEN, Beijing, China), 1 μL of the forward and reverse primers (0.5 μM each), 3 μL of RNA template (diluted to 100 ng/μL), and 5.5 μL of RNase-free water [10]. Cycling conditions were as follows: 50 °C for 30 min; 95 °C for 5 min followed by 35 cycles of 95 °C for 15 s, 50 °C for 30 s, and 72 °C for 2 min; a final extension of 5 min at 72 °C. The following primers used for detecting the 10 nasal swab samples were as follows: F: 5′-CCCTATAATTATTTGGGTTT CTCCTTAT-3′, R: 5′-AGTGACGAGTTGATTCTCCAGTATAG-3′. To obtain the complete sequence of ENTV-2, a total of 6 pairs of primers (Table 1) were designed and synthesized according to ENTV-2 sequences that were deposited in the GenBank database (accession number: MK210250, MT254063, MK164400). The PCR products were purified by a Universal DNA Purification Kit (TIANGEN, China). Subsequently, the recovered PCR products were cloned into the pMD-19T vector (Takara, China), and the Sanger method was used for DNA sequencing.

### 2.4. Sequence Assembly and Phylogenetic Analysis

The obtained nucleotide sequences by DNA sequencing were submitted for BLAST analysis to verify that they were from ENTV-2. Then, the SeqMan program in the DNASTAR Lasergene v7.1 software package (Madison, WI, USA) was used for assembling the full-length genome of ENTV-2. Nucleotide sequence editing, analysis, and alignments were conducted by the corresponding programs in the DNASTAR v7.1 software package (DNASTAR Inc. Madison, WI, USA). Phylogenetic analysis of ENTV-2 based on the complete sequences was performed by neighbor-joining method using MEGA v7.1 software with 500 bootstrap replicates [20].

### 2.5. Sequence Annotation and Comparative Analysis of ENTV-2 Whole Genome Sequences

The CQ2 strain was annotated based on the annotation of other complete ENTV-2 sequences in the GenBank database. Then, the ENTV-2 CQ2 genome was aligned with those of the representative isolates FJ/Fujian/2019 (accession number: MK559457), BH/Guangxi/2019 (accession number: MT254062), Shaanxi2/Shaanxi/2015 (accession number: KU980910), CQ1/Chongqing/2018 (accession number: MK164400), CHN2/Sichuan/2013 (accession number: KU258871), GDQY2017/Guangdong/2017 (accession number: MK164396), CHN5/Sichuan/2013 (accession number: KU258874), CHN8/Sichuan/2013 (accession number: KU258877), and CHN9/Sichuan/2013 (accession number: KU258878). The comparative analysis was performed by using the mVista LAGAN genomics analysis tool (https://genome.lbl.gov/vista/index.shtml, accessed on 8 November 2023) [21,22].

### 2.6. Recombination Analysis

In total, 17 complete sequences of ENTV-2 from the GenBank database (Table S1) and CQ2 in this study were aligned by the MAFFT online server (https://mafft.cbrc.jp/alignment/server/, accessed on 24 November 2023) with default parameters, and then the alignment result was analyzed by using two distinct software tools. The RDP v4.39 software was utilized to detect potential inter-strain recombination events and determine potential breakpoint locations. The default parameters were applied, and a Bonferroni-corrected threshold of *p* = 0.05 was set. The SimPlot v3.5.1 program was utilized to identify the putative recombinant and further confirm the recombination events, which performed the bootscan analysis with a sliding window of 400 bp, a step size of 40 bp, gap strip, and the Kimura 2-parameter substitution model [20]. Phylogenetic trees were then constructed to validate the detected recombination signals when querying strain CQ2, using the alignments of recombination region and non-recombination region divided by the predicted locations of breakpoints, respectively.

## 3. Results

### 3.1. Molecular Detection and Genomic Sequencing of ENTV-2

**Figure 1:**
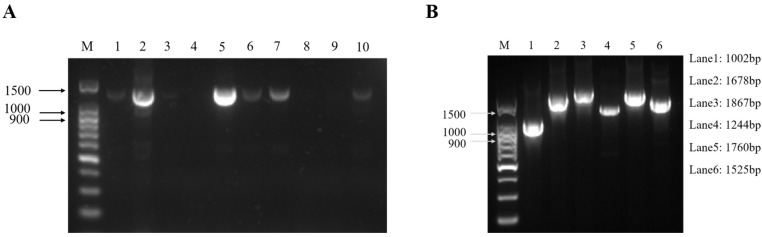
Electrophoresis patterns of the fragment of ten nasal swab samples from suspected ENA cases (**A**) and the six fragments of ENTV-2 CQ2 (sample 5) that were amplified by one-step RT-PCR (**B**). (**A**) Lane M was the DNA marker; lanes 1–10 were nasal swab samples from suspected cases. The positive band with a size of 1372 bp was observed after agarose gel electrophoresis. Two of the ten samples were strongly positive (sample 2 and sample 5) and four were weakly positive (sample 1, sample 6, sample 7, and sample 10). (**B**) Lane M was the DNA marker; lanes 1–6 were six different fragments, and the corresponding sizes of the six fragments are shown on the right side.

In April 2022, suspected ENA cases were found on a goat farm in Chongqing. Several goats developed respiratory symptoms and there were no deaths. The nasal fluids of ten goats with significant respiratory symptoms were collected. Then, RNA was extracted and used for a one-step reverse transcription PCR. The results showed that two samples were strongly positive and four samples were weakly positive among the ten samples (Figure 1A). Among them, sample 5 was the strongest positive sample, so it was selected for whole-genome fragment amplification and the subsequent studies by segmented PCR method. The results showed that six fragments were obtained by segmented PCR amplification (Figure 1B). After TA cloning and Sanger sequencing, the complete genome sequence of one ENTV-2 isolate in this study was obtained with 7468 bp in length and designated as CQ2. The complete genome sequence of CQ2 has been submitted into the GenBank database with accession number OR682176.

### 3.2. Phylogenetic Analysis of ENTV-2 CQ2

Multiple alignments based on the complete genome sequences of ENTV-2 showed that CQ2 shared high similarity with other Chinese ENTV-2 strains at the nucleotide sequence level. To further understand the genetic variation in ENTV-2 CQ2, we conducted a phylogenetic analysis based on the complete genome sequences of ENTV-2, Jaagsiekte sheep retrovirus (JSRV), and endogenous JSRV (enJSRV) representative strains by neighbor-joining method using MEGA v11 software.

**Figure 2:**
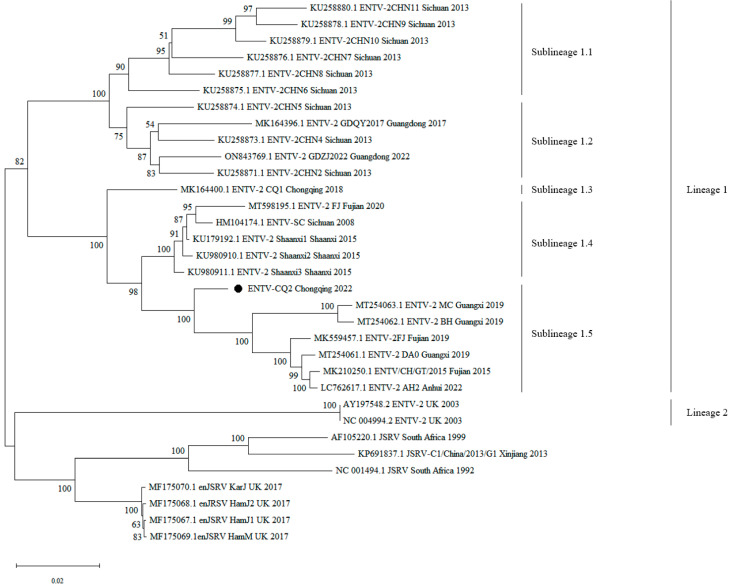
Phylogenetic tree construction based on the complete genome sequences of ENTV-2 and its related viruses. Phylogenetic tree was conducted by the neighbor-joining method using MEGA v11 software with 500 bootstrap replicates. Bootstrap values of more than 50% were shown on the corresponding nodes. Black circle indicates the virus investigated in the current study. GenBank accession numbers along with strain names, collection sites, and years are indicated for the corresponding viruses.

The results showed that all ENTV-2 strains of China clustered together in one large branch, while ENTV-2 strains of the UK, JSRV, and enJSRV clustered in another large branch. Additionally, ENTV-2 exhibited a certain degree of genetic diversity and was typically geographically clustered (Figure 2). As shown in the phylogenetic tree, the ENTV-2 strains could be divided into two main lineages: lineage 1 was composed of all the Chinese strains; lineage 2 was composed of strains from other countries. Furthermore, lineage 1 could be subdivided into 5 sublineages, of which sublineages 1.1, 1.2, 1.3, and 1.4 have been mentioned and reported in our previous paper [10]. Sublineage 1.5 was a newly formed sublineage, which was mainly composed of CQ2 from Chongqing and other recently emerged strains from Guangxi, Fujian, and Anhui (Figure 2).

Additionally, the ENTV-2 strains from lineage 1 exhibited a certain degree of spatial and temporal clustering. In terms of temporal distribution, compared to the other four sublineages, the strains of sublineage 1.5, including CQ2, emerged slightly later in time. In terms of the spatial distribution of strains, the strains in sublineage 1.1 were primarily from Sichuan Province, those in sublineage 2 were primarily from Sichuan and Guangdong provinces, those in sublineage 1.3 were from Chongqing, those in sublineage 1.4 were primarily from Sichuan and Fujian provinces, and the newly emerged strains in sublineage 1.5 were primarily from Guangxi, Fujian, Anhui, and Chongqing, and CQ2 was closely related to the Guangxi and Fujian strains. These findings suggested that the Chinese ENTV-2 isolates exhibited a geographic clustering pattern. Furthermore, lineage 1 was composed entirely of Chinese strains, while lineage 2 was composed of strains from the UK, further indicating that the phylogeny of ENTV-2 is correlated with its geographical distribution (Figure 2).

### 3.3. Comparative Genomic Analysis of ENTV-2 Strains

**Figure 3:**
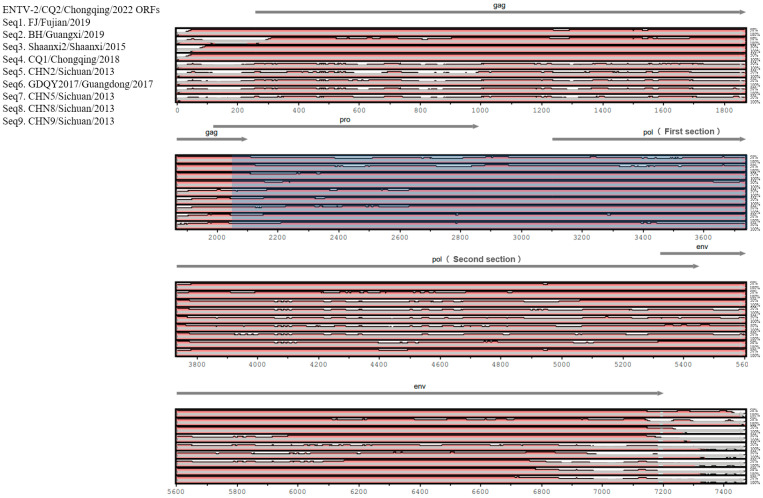
Genomic organization of ENTV-2 CQ2 and sequence conservation comparison with the FJ/Fujian/2019, BH/Guangxi/2019, Shaanxi2/Shaanxi/2015, CQ1/Chongqing/2018, CHN2/Sichuan/2013, GDQY2017/Guangdong/2017, CHN5/Sichuan/2013, CHN8/Sichuan/2013, and CHN9/Sichuan/2013 isolates. Sequence conservation was determined from a multiple sequence alignment.

In terms of genomic composition, the genome of CQ2 was consistent with other ENTV-2 strains, which were composed of the following four genes: *gag*, *pro*, *pol*, and *env*. To compare ENTV-2 strains from different geographical locations in China, we evaluated the sequence diversity among CQ2 and the selected nine genome sequences from its closely related sublineages. We found that the *gag* and *env* gene regions of CQ2 were highly similar to those of strains FJ/Fujian/2019, BH/Guangxi/2019, Shaanxi2/Shaanxi/2015, and CQ1/Chongqing/2018 from sublineages 1.3, 1.4 and 1.5. However, these sequence regions were significantly different from those of strains CHN2/Sichuan/2013, GDQY2017/Guangdong/2017, CHN5/Sichuan/2013, CHN8/Sichuan/2013, and CHN9/Sichuan/2013 from sublineage 1.1 and sublineage 1.2 (Figure 3). In contrast, the *pro* and the first section of the *pol* gene regions of CQ2 were more similar to the corresponding regions of CHN2/Sichuan/2013, GDQY2017/Guangdong/2017, CHN5/Sichuan/2013, CHN8/Sichuan/2013, and CHN9/Sichuan/2013 from sublineages 1.1 and 1.2. However, the second section of the *pol* gene showed higher similarity to the corresponding gene regions of FJ/Fujian/2019 and BH/Guangxi/2019 from sublineage 1.5 (Figure 3). This suggested that the CQ2 genome may be derived from recombination between strains from different sublineages.

### 3.4. Recombination Analysis among ENTV-2 Strains

**Figure 4:**
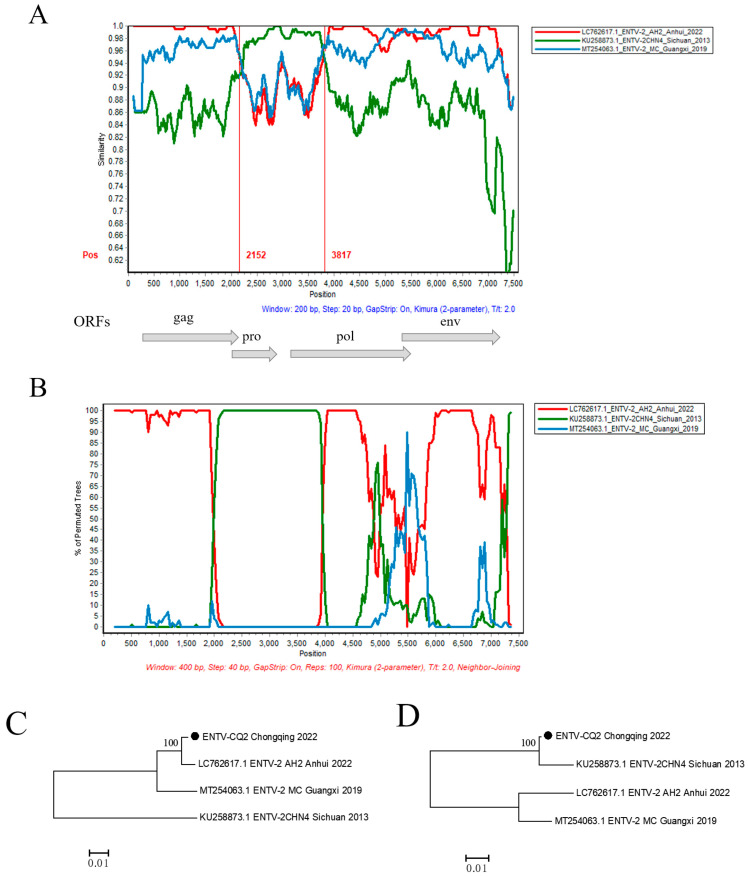
Recombination analysis of the CQ2 strain. (**A**) Genome-scale similarity comparisons of strain CQ2 against strains CHN4, AH2, and MC. (**B**) BootScan analysis in the SimPlot software was performed with CQ2 as the query sequence, CHN4 and AH2 as putative parental isolates, and MC as the outgroup isolate. (**C**,**D**) Phylogenetic trees of non-recombination region (the corresponding loci in the alignment: 1–2152; 3817–terminal site) and recombination region (the corresponding loci in the alignment: 2153–3816) are shown, respectively. The phylogenetic trees were constructed using the neighbor-joining (NJ) method in MEGA v11 software with 1000 bootstrap replicates.

In order to further validate the possible recombination events and breakpoint positions in the ENTV-2 CQ2 strain, the full-length genomic sequences of the CQ2 strain and its 17 potential parental sequences were analyzed by RDP v4.39 and SimPlot v3.5.1 software (Table S1). The results showed that CQ2 might be a natural recombinant with CHN4/Sichuan/2013 (Sublineage 1.2) as the minor parent and AH2/Anhui/2022 (Sublineage 1.5) as the major parent. Through analysis of the similarity plot and bootscan plot performed by the SimPlot v3.5.1 software, we detected a potential recombination region on the analyzed sequence with two distinct recombination breakpoints at positions 2152 nt and 3817 nt, respectively (Figure 4A,B). In order to verify the recombination signals detected in CQ2, we conducted phylogenetic analyses of the regions separated by recombination breakpoints, including both recombinant and non-recombinant regions. The phylogenetic tree based on the non-recombinant region showed that AH2/Anhui/2022 from sublineage 1.5 and CQ2 were closely clustered on an independent branch. However, the phylogenetic tree based on the recombinant region showed that CHN4/Sichuan/2013 from Sublineage 1.2 and CQ2 were closely clustered on one relatively independent branch, which showed conflicting phylogenetic relationships compared to the phylogeny based on non-recombinant region (Figure 4C,D).

Furthermore, the unique single nucleotide polymorphisms (SNPs) in the recombinant region shared by CHN4 and CQ2 that could lead to substitutions of their corresponding amino acid were detected and analyzed. The results showed that a total of 20 SNPs in the recombinant region resulted in 15 amino acid substitutions. Specifically, 11 SNPs were scattered in the *pro* gene and resulted in seven amino acid substitutions; and nine SNPs in the *pol* gene resulted in eight amino acid substitutions (Table 2). Hence, we concluded that a recombination event might occur in the corresponding regions of *pro* and *pol* genes between sublineage 1.5 and sublineage 1.2 strains.

## 4. Discussion

The ENA of goats is a highly lethal infectious disease caused by ENTV-2 infection. This virus causes chronic, progressive, and contact diseases in goats. The clinical symptoms include loss of appetite, extreme thinness, copious serous nasal discharge, followed by progressive dyspnea, and ultimately death from suffocation. Recent studies indicated that the incidence of ENA in China showed an upward trend, and its spreading areas were also expanding. In recent years, ENA has been reported in multiple provinces in China, including Shaanxi, Anhui, Sichuan, Chongqing, Fujian, Guangxi, Guangdong, and Inner Mongolia [23]. Hence, the genetic variation and evolution of ENTV-2 in China need to be further investigated.

In our study, we collected nasal fluid samples of suspected ENA cases from the goat farm in Chongqing, conducted PCR detection, and then sequenced and analyzed the whole genome of one ENTV-2 strain (CQ2). The complete sequence of ENTV-2 CQ2 obtained by Sanger sequencing was 7468 bp in length, which had been submitted to GenBank with the accession number OR682176. Phylogenetic analysis suggested that the ENTV-2 strains were geographically clustered. It showed that the ENTV-2 strains could be divided into two main lineages: lineage 1 was composed of all the Chinese strains; lineage 2 was composed of strains from other countries. Similar to other Chinese strains, CQ2 also belonged to lineage 1 and was further classified into sublineage 1.5 (Figure 2).

In July 2018, we reported the occurrence of one ENTV-2 strain (ENTV-2 CQ1, accession number: MK164400) in the goat population of Chongqing [10]. This was the first report of ENA in the goat population of Chongqing. However, five years later, the CQ2 strain isolated from Chongqing exhibited a distant genetic relationship with the CQ1 strain. As shown in the phylogenetic tree, the two strains were in two independent branches, with CQ1 in a separate branch of sublineage 1.3 and CQ2 in the evolutionary branch of sublineage 1.5. The sublineage 1.5 seemed to be a new sublineage, which consisted of CQ2 and its closely related strains from Guangxi, Fujian, and Anhui. This finding indicated that the ENTV-2 strains in Chongqing continued to undergo genetic variation since CQ1 was reported. Furthermore, the development of the transportation and logistics industry has been considered to facilitate the transmission of ENTV-2 between different regions and accelerate the process of genetic mutation [16]. Considering the close genetic relationship between CQ2 and strains in other provinces of China, it is suggested that the occurrence of CQ2 may be attributed to the circulation of goats between different provinces of China.

Furthermore, the sequence diversity among CQ2 and the nine strains from its closely related sublineages showed that the *gag* and *env* gene regions of CQ2 were highly similar to those of the strains from sublineages 1.3, 1.4, and 1.5; however, these sequence regions were significantly different from those of strains from sublineage 1.1 and sublineage 1.2 (Figure 3). In contrast, the *pro* and part of the *pol* gene regions of CQ2 were more similar to the corresponding regions of strains from sublineages 1.1 and 1.2 than to those of strains from sublineage 1.5 (Figure 3). This suggested that the CQ2 genome may be derived from recombination between the strains of different sublineages.

To further investigate the origin of gene variation in ENTV-2 CQ2, the recombination analysis based on the complete genome sequences of ENTV-2 was conducted. It was found that CQ2 may be a recombinant between ENTV-2 strains from sublineage 1.2 and sublineage 1.5. Furthermore, it was indicated that AH2 from Anhui province could be the major parental strain of CQ2, and CHN4 from Sichuan province could be the minor parental strain. The genome-scale similarity analysis revealed that the recombination region was situated within the *pro* and *pol* gene region (2152 nt-3817 nt), thereby implying the occurrence of recombination events within this specific genomic area (Figure 4). Through this study, we not only gained insights into the evolutionary relationships of the newly emerged ENTV-2 in Chongqing but also found that this virus may be a previously unreported recombinant strain.

The protein encoded by the *pro* gene may consist of two structures: one is a protease with deoxyuridine triphosphate activity, which is also found in other retroviruses [24]. The other is an active protease (PR), and Leu-Asp-Thr-Gly may be the core amino acid sequence of PR [25]. The *pol* gene mainly encodes the highly conserved reverse transcriptase (RT). It is speculated that its active site encodes a sequence of Tyr-Met-Asp-Asp [25]. RT has a variety of enzyme activities, and the main specific enzyme activities are polymerase activity and RNase H activity. Reverse transcriptase activity utilizes RNA as a template to synthesize complementary DNA. RNase H activity can hydrolyze RNA in heterozygous duplex to obtain DNA single strand (cDNA) complementary to RNA [26]. Interestingly, the recombinant regions in this study were mainly located in the *pro* and *pol* genes and corresponded to the positions of viral encoded PR and RT (Table 2). Furthermore, there are 15 amino acid substitutions in PR and RT (Table 2), which may affect the corresponding functions of the two proteins. Future studies are needed to determine whether the recombination occurring in the *pro* and *pol* gene regions has an impact on viral virulence and infection. In contrast, no evidence of recombination was found in the *gag* and *env* genes. The structural proteins encoded by the *gag* gene ensure the accurate positioning of virus particle assembly, while the envelope proteins encoded by the *env* gene possess a high degree of specificity in binding with the host, playing a pivotal role in mediating the entry of virus particles into the cells [27,28,29,30]; all of these may potentially confer higher conservation and lower mutation rate of *gag* and *env* [31,32].

In conclusion, our study summarized the evolutionary relationships of the newly emerged ENTV-2 strain CQ2 in Chongqing and further revealed that this strain was a previously unreported recombinant strain within the prevalent Chinese strains. The prerequisite for virus recombination is that two or more parental strains co-infect the same host–cell and exchange genetic material to produce a recombinant [33]. Therefore, this suggested that as a retrovirus, ENTV-2 may have undergone cross-infection in the goat population, leading to the emergence of new variants. However, whether new ENTV-2 variants, such as CQ2, will have greater virulence or immune evasion remains to be explored. The findings in this study will contribute to a better understanding of the transmission and evolution of ENTV-2, providing a valuable reference for future research and prevention and control efforts.

## 5. Conclusions

This study provides insights into the genetic evolution and emergence of a novel recombinant ENTV-2 strain (CQ2) in goats in Chongqing, China. The phylogenetic analysis reveals that ENTV-2 strains cluster geographically and CQ2 belongs to sublineage 1.5, exhibiting a distant genetic relationship with a previous strain CQ1 from Chongqing. The sequence analysis suggests that CQ2 is a recombinant strain derived from sublineages 1.2 and 1.5. These findings will enhance understanding of the transmission and evolution of ENTV-2, providing an important reference for the prevention and control of ENTV-2 infection.

## Figures and Tables

**Table 1:** Primers used for enzootic nasal tumor virus type 2 (ENTV-2) genome amplification in this study.

Primer	Sequence (5′-3′)	Position in CQ2 (OR682176) (bp)	Product Length (bp)
ENTV-2-1-F	ACAAGGCATCAGCCATTTTGGT		
ENTV-2-1-R	AACCTCACCAAGTCGCTGC	1–1002	1002
ENTV-2-2-F	GGGGAACAAATTCGAACTCAT TATACT		
ENTV-2-2-R	CCCATCTCAGGTGCTAGTATT GTAT	433–2110	1678
ENTV-2-3-F	GCAACCCTGCAGGTTCCAA		
ENTV-2-3-R	TGTGGGTGCTCGAGGGGTA	1999–3865	1867
ENTV-2-4-F	ATTGCTGATGAAAAAATT		
ENTV-2-4-R	TATTGAGGGAGAACAAA	3497–4740	1244
ENTV-2-5-F	GCAAATGATTGAAACTGT		
ENTV-2-5-R	ACTATTGCCATGACCAAA	4393–6152	1760
ENTV-2-6-F	CTCCTTGGACTTTATGTCGAGC		
ENTV-2-6-R	TGTTTTATTGTGTCATAGTATA	5944–7468	1525

**Table 2:** Detection of unique SNPs and their resulting amino acid substitutions shared by CHN4 and CQ2 in the recombination region.

Gene	Loci in the Genome of CQ2	CQ2	CHN4	AH2	MC
*pro*	2273	C ^a^ (K) ^b^	C (K)	T (R)	T (R)
	2369	A (E)	A (E)	T (D)	T (D)
	2503–2504	AA (E)	AA (E)	GT (G)	GT (G)
	2608–2609	GG (R)	GG (R)	CA (T)	CA (T)
	2738	A (E)	A (E)	T (D)	T (D)
	2799	A (I)	A (I)	G (V)	G (V)
	2829–2831	AGT (S)	AGT (S)	GAG (E)	GAG (E)
*pol*	3176	A (I)	A (I)	G (V)	G (V)
	3294	G (R)	G (R)	A (K)	A (K)
	3356	T (L)	T (L)	A (I)	A (I)
	3461	A (I)	A (I)	G (V)	G (V)
	3494	G (V)	G (V)	A (I)	A (I)
	3555	A (Y)	A (Y)	T (F)	T (F)
	3564–3565	TT (V)	TT (V)	CC (A)	CC (A)
	3746	T (S)	T (S)	C (P)	C (P)

^a^ refers to the corresponding nucleotide in the indicated position. ^b^ refers to the substituted amino acid by SNP in the indicated position.

## Data Availability

The data presented in this study are available in the article.

## Supplementary Materials

The following supporting information can be downloaded at: https://www.mdpi.com/article/10.3390/vetsci11060248/s1, Table S1. Information of the 17 ENTV-2 strains from GenBank for recombination analysis.
