# Supplementary material for: Genomic Sequencing and Analysis of Enzootic Nasal Tumor Virus Type 2 Provides Evidence for Recombination within the Prevalent Chinese Strains
Source: Vet Sci. 2024 Jun 2;11(6):248. doi: 10.3390/vetsci11060248 (PMC11209414; doi:10.3390/vetsci11060248)
Supplement: Supplementary file 1 [file vetsci-11-00248-s001.zip › vetsci-2994430-supplementary.pdf]

Table S1. Information of the 17 ENTV-2 strains from GenBank for recombination analysis.

Accession number	Name	Country/region	Year
LC762617.1	ENTV-2 AH2	China, Anhui	2022
MK210250.1	ENTV/CH/GT/2015	China, Fujian	2015
MK559457.1	ENTV-2 FJ	China, Fujian	2019
MT254062.1	ENTV-2 BH	China, Guangxi	2019
MT254061.1	ENTV-2 DA0	China, Guangxi	2019
MT254063.1	ENTV-2 MC	China, Guangxi	2019
MK164396.1	ENTV-2 GDQY2017	China, Guangdong	2017
ON843769.1	ENTV-2 GDZJ2022	China, Guangdong	2022
KU258871.1	ENTV-2CHN2	China, Sichuan	2013
KU258873.1	ENTV-2CHN4	China, Sichuan	2013
KU258874.1	ENTV-2CHN5	China, Sichuan	2013
KU258875.1	ENTV-2CHN6	China, Sichuan	2013
KU258876.1	ENTV-2CHN7	China, Sichuan	2013
KU258877.1	ENTV-2CHN8	China, Sichuan	2013
KU258878.1	ENTV-2CHN9	China, Sichuan	2013
KU258879.1	ENTV-2CHN10	China, Sichuan	2013
KU258880.1	ENTV-2CHN11	China, Sichuan	2013
